# The Role of Focused Echocardiography in Pediatric Intensive Care: A Critical Appraisal

**DOI:** 10.1155/2015/596451

**Published:** 2015-10-28

**Authors:** Heloisa Amaral Gaspar, Samira Saady Morhy

**Affiliations:** ^1^Pediatric Intensive Care Unit, Albert Einstein Hospital, Rua do Carreiro de Pedra 111, Apartamento 152 C, 04728-020 São Paulo, SP, Brazil; ^2^Echocardiography Department, Albert Einstein Hospital, Rua Pintassilgo 458, Apartamento 53, 04514-032 São Paulo, SP, Brazil

## Abstract

Echocardiography is a key tool for hemodynamic assessment in Intensive Care Units (ICU). Focused echocardiography performed by nonspecialist physicians has a limited scope, and the most relevant parameters assessed by focused echocardiography in Pediatric ICU are left ventricular systolic function, fluid responsiveness, cardiac tamponade and pulmonary hypertension. Proper ability building of pediatric emergency care physicians and intensivists to perform focused echocardiography is feasible and provides improved care of severely ill children and thus should be encouraged.

## 1. Introduction

Echocardiography is currently considered a key tool for the hemodynamic assessment in Intensive Care Units (ICU), able to identify causes of hemodynamic instability and to quickly guide therapy [1, 2]. Some of its advantages are being a noninvasive method, risk-free, capable of being performed serially and in real time, and analyzed along with clinical data by intensivists.

Several studies have demonstrated the positive effect of the use of echocardiography in the management of critically ill patients, changing their treatment in 30%–60% of cases after the test is performed [3–6]. Thus, the recent expert consensuses and reviews on shock point out the importance of echocardiography in the identification of the pathophysiology and categorization of shock as distributive, hypovolemic, obstructive, or cardiogenic [7].

In the pediatric age range, there is an important limitation of noninvasive devices for hemodynamic monitoring, and this makes the use of echocardiography even more promising. An interesting review on hemodynamic monitoring suggests using focused echocardiography with the monitoring devices already routinely used to assess the hemodynamic status of critically ill children [8].

When compared to the full echocardiography, the purpose of the focused echocardiography is the early identification of limited hemodynamics changes thus expediting clinical decisions regarding treatment [9, 10].

Although there is no consensus on the ideal training format for capacity building of nonechocardiographers in focused echocardiography, different training programs for physicians from different areas (anesthetists, internists, intensivists, surgeons, and pediatricians) to perform specific echocardiographic assessments have been published [5, 11–21]. In a previous study, we demonstrated that pediatricians specialized in Emergency or Intensive Care are able to perform focused echocardiography with a good concordance when compared to experienced echocardiographers, after 10 hours of theoretical sessions and 24 real-time training exams performed under supervision [22].

The most relevant parameters assessed by focused echocardiography in pediatrics are left ventricular systolic function, volemia/response to fluid resuscitation, pericardial effusion/cardiac tamponade, and right ventricular systolic function and pulmonary hypertension.

## 2. Left Ventricular Systolic Function

Previous studies have demonstrated the inaccuracy of physical examination in the assessment of the cardiac function and hemodynamic profile of critically ill patients, even when performed by an experienced physician [23]. The echocardiographic assessment of the left ventricular (LV) function as an extension of physical examination has a proven beneficial effect on the timing to start therapy as well as on its quality. A study conducted in adult patients with congestive heart failure showed a significant improvement in the identification of patients with severe LV dysfunction when the medical assessment was associated with focused echocardiography, and this was the main factor for the identification of this group of patients (OR = 154, *p* < 0.001) [24]. Similar findings were reported in an Intensive Care environment, where the use of echocardiography by the intensivist brought additional information regarding the cardiac function and provided changes in therapy in 37% of the patients assessed [5].

In a Pediatric Intensive Care Unit, Ranjit et al. [6] suggested how the echocardiographic analysis of LV systolic function may be part of a multimodal hemodynamic assessment in association with physical examination and invasive blood pressure monitoring and thus may be incorporated in the medical arsenal to care for children with septic shock. In their study, the authors identified the presence of cardiac dysfunction echocardiographically in 45% of children after the baseline medical approach and demonstrated a favorable outcome in 91.6% of cases when the therapeutic management was guided by multimodal hemodynamic monitoring.

The left ventricular systolic function may be assessed by echocardiography both qualitatively and quantitatively.

### 2.1. Qualitative Analysis of the LV Systolic Function

The qualitative analysis of the LV systolic function consists of the visual analysis of the examiner in relation to the myocardial contractile function and is the method of choice for the assessment of the LV function by nonechocardiographers [25].

Left ventricular ejection fraction (EF) is estimated visually using multiple echocardiography views: the parasternal long and short views and the apical and the subcostal views. This assessment is made by analyzing the myocardial thickness during systole and the reduction of the ventricular chamber diameter during systole in comparison to diastole provided by the ventricular wall motion during systole. LV function is subjectively classified as normal (EF ≥ 55%), slightly reduced (EF 41%–55%), moderately reduced (EF 31%–40%), and markedly reduced (EF ≤ 30%) [26].

### 2.2. Quantitative Analysis of the LV Systolic Function

The quantitative analysis of the LV systolic function consists of LV ejection fraction and cardiac output/index measurements. This data aids the physician in choosing between therapeutic options, like fluids and/or inotropic agents.

**Figure 1:**
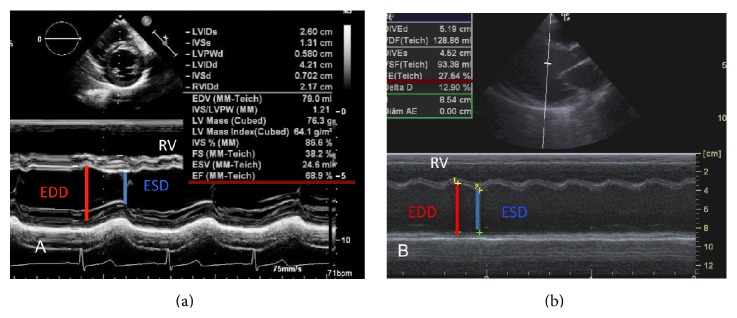
Calculation of left ventricular fractional shortening by the M mode. (a) Parasternal short-axis view in a patient with normal ejection fraction. (b) Parasternal long-axis view in a patient with viral myocarditis and cardiogenic shock, with reduced ejection fraction. EDD: left ventricle end-diastolic diameter; ESD: left ventricle end-systolic diameter; RV: right ventricle. FS = EDD − ESD/EDD × 100.

Ejection fraction may be measured in the M mode or two-dimensional mode. EF calculation in the M mode is the most widely used in clinical practice, especially in pediatric patients, and is derived from the fractional shortening (FS) measurement. Measurements of the LV systolic (ESD) and diastolic diameter (EDD) right below the mitral valve leaflets in the parasternal short- or long-axis views are necessary to obtain the fractional shortening, which is calculated using the formula FS = EDD − ESD/EDD × 100 (Figure 1).

The clinical usefulness of quantitative EF measurements in the management of critically ill patients is broadly accepted both in adult and in pediatric patients [1, 27]; however, it is important to emphasize that this assessment should not be used without also considering patient's preload, cardiac output, and tissue perfusion in order to minimize the improper management of inotropic agents [28]. Studies show that trained nonechocardiographers are able to perform the quantitative analysis of EF even in pediatric patients and that this information may be positively added to the care of hemodynamically unstable pediatric patients [6, 13, 22].

Similar to the qualitative assessment, EF is classified as normal (EF ≥ 55%), slightly reduced (EF 41%–55%), moderately reduced (EF 31%–40%), and markedly reduced (EF ≤ 30%) [26].

The cardiac output (CO) evaluation is not recommended to all shock patients. However, a recent experts consensus was proposed as evidence level I that the CO measurement should be performed in those patients that are not responding to initial therapy to evaluate the response to fluids or inotropes [28]. As previously cited, it is crucial that the CO assessment is not the only variable used to decide treatment but is indeed added in the preload, left ventricular ejection fraction, and tissue perfusion equation. This concept is supported by a prior trial performed by Vieillard-Baron et al. that demonstrated the existence of patients with low ejection fraction but normal cardiac index, as well as patients with low cardiac index and normal ejection fraction [1]. Hence, inotropic agents should only be given when the compromised ejection fraction is accompanied by inadequate cardiac output and tissue hypoperfusion.

**Figure 2:**
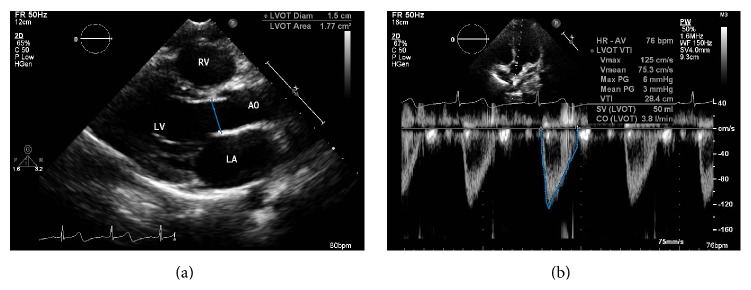
Stroke volume (SV) calculation. (a) Measurement of LV outflow tract diameter (LVOTD) using the parasternal long-axis view, and (b) use of pulsed Doppler for the measurement of velocity-time integral (VTI), as obtained in the 5-chamber apical view. Cardiac output (CO) = SV × HR; SV = VTI × LVOT area, where LVOT area =  *π*(LVOT  diameter/2)^2^.

The CO measurement by echocardiography depends on a combination of measurements made in the two-dimensional mode and aortic blood flow study by Doppler. CO is calculated by multiplying the stroke volume (SV) by the heart rate (HR). SV is obtained by measuring the LV outflow tract (LVOT) diameter in the two-dimensional mode (parasternal long-axis view) and the velocity-time integral (VTI) by pulsed Doppler (5-chamber apical view), with SV = VTI × LVOT area, where LVOT  area = *π*(LVOT  diameter/2)^2^ (Figure 2). The targeted cardiac index in septic children suggested by Surviving Sepsis Campaign is between 3.3 and 6.0 L/min/m^2^ [29].

For requiring the use of Doppler, CO measurement may be deemed technically challenging for the nonspecialist physician. Nonetheless, in a previous study conducted in pediatric patients [22], we demonstrated that it is possible to train pediatricians for the analysis of CO, like in a study conducted in adult patients [30], however pioneering in pediatrics. This assessment may provide a new option for hemodynamic monitoring of severely ill children, a population that lacks noninvasive methods for CO measurement.

## 3. Fluid Responsiveness and Preload Estimation

Fluid resuscitation is part of the initial management of shock. However, aggressive fluid resuscitation may be harmful to some patients and some types of shock [31]. Assessment of the preload and fluid responsiveness is key in the management of critically ill patients and previous pediatric studies demonstrated that only 40–69% of children responded to intravascular volume expansion [32–34]. Clinical assessment and static measurements of filling pressures (central venous pressure and pulmonary wedge pressure) did not predict fluid responsiveness in children, which is consistent with findings in adults [35, 36]. However, in contrast to adults, dynamic variables as pulse pressure variation and stroke volume variation also did not predict fluid responsiveness in children, and that makes this evaluation in pediatric patients even more challenging [36].

**Figure 3:**
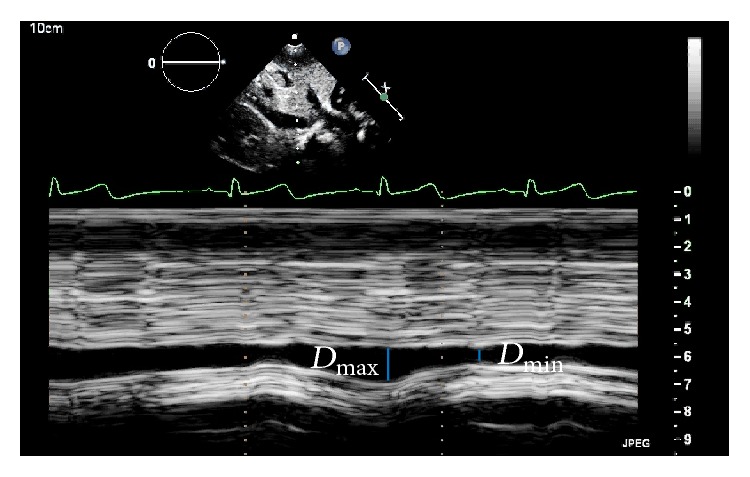
M mode echocardiography from subcostal view in a five-year-old patient in septic shock for urinary tract infection under mechanical ventilation, with sustained hypotension after volume expansion with 60 mL/kg of saline solution. Bedside echocardiography showed significant respiratory changes in IVC diameter, which, along with other clinical and monitoring data, suggested that fluid resuscitation should be maintained. dIVC = 90%, where dIVC = (*D*_max_ − *D*_min_)/*D*_min_  × 100.

The first form of assessing preload and fluid responsiveness by echocardiography is by analyzing the inferior vena cava (IVC) diameter. However, the static measurement of IVC diameter has a poor correlation with the patient's individual response to fluid resuscitation, especially in children in whom the IVC diameter is related to their weight and height [37]. Respiratory changes in IVC diameter are the most frequently used echocardiographic method for the assessment of the fluid responsiveness and consist of the analysis of the IVC diameter change with respiration while under positive pressure ventilation (inspiration and expiration) (Figure 3).

Respiratory changes in IVC by transthoracic echocardiography were established in the clinical practice after two studies conducted in adult patients undergoing mechanical ventilation. These studies showed a strong correlation between respiratory change in IVC and the patient's fluid responsiveness [38, 39]. The authors showed a linear correlation between respiratory changes in IVC and increased CO after fluid loading, where the greater the respiratory change in IVC prior to fluid replacement, the higher the increase in CO after fluid replacement. The index described by Barbier et al. [39], called inferior vena cava distensibility index (dIVC) and calculated using the formula dIVC = (*D*_max_ − *D*_min_)/*D*_min_  × 100, showed the best cutoff value of 18% to discriminate volume-responsive individuals (dIVC > 18%) from non-volume-responsive individuals (dIVC < 18%) with 90% sensitivity and specificity. However, we should note that it was performed in sedated patients with normal sinus rhythm, no spontaneous breathing, and a tidal volume of 10 mL/kg under mechanical ventilation. For having the advantage of being a noninvasive method that can be performed quickly and serially, the use of dIVC became widespread, but the specific situation in which the study was conducted cannot be disregarded [40]. There are few studies correlating fluid responsiveness with respiratory changes in IVC in pediatric patients and these studies have shown conflicting results; therefore, this is an open field for further investigations [41, 42].

Another means of assessing fluid responsiveness using echocardiography is by analyzing the respiratory change of the aortic peak flow velocity on Doppler during inspiration and expiration in patients under mechanical ventilation. It is calculated as Δ*V* = (*V*_peak  max_ − *V*_peak  min_)/*V*_mean  peak_ [43]. The aortic peak flow variation has been correlated with the patient's response to fluid infusion in several previous studies, including at least five studies with pediatric patients, and emerges as a promising form of assessment of fluid responsiveness in children under mechanical ventilation [34, 41, 42, 44, 45].

## 4. Pericardial Effusion and Cardiac Tamponade

Pericardial effusion (PE) is identified in echocardiography as an echolucent space adjacent to the cardiac structures. It may be diffuse or loculated, more frequently is diffuse and promote a clear separation between the parietal and visceral pericardia [46]. Loculated effusion may be secondary to adherences after cardiac surgery or trauma.

**Figure 4:**
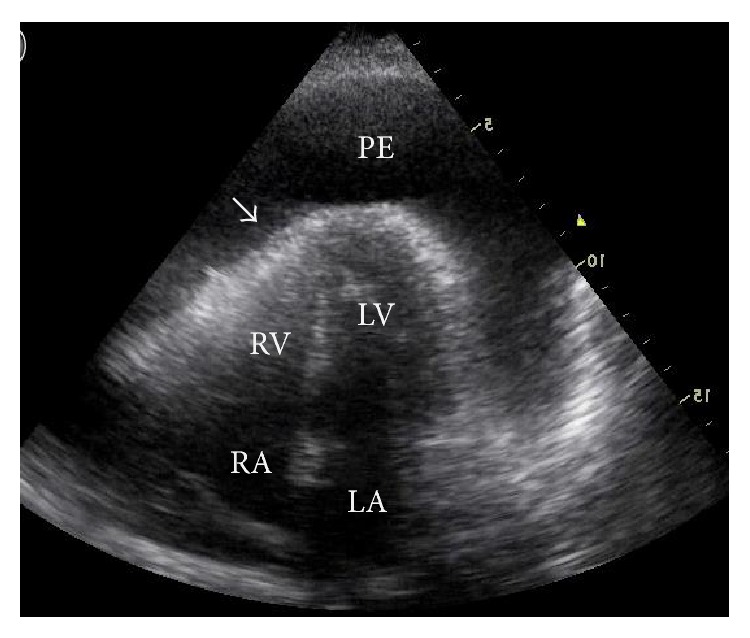
Cardiac tamponade with large pericardial effusion and diastolic collapse of the right ventricle (arrow). LA: left atrium; LV: left ventricle; PE: pericardial effusion. RA: right atrium; RV: right ventricle.

Cardiac tamponade is an emergency situation and its diagnosis is known to be clinical. However, echocardiography may suggest the existence of tamponade physiology, that is, increased intrapericardial pressure precluding the ventricular filling. The major echocardiographic signs that corroborate the clinical diagnosis of cardiac tamponade are systolic collapse of the right atrium and diastolic collapse of the right ventricle, presence of IVC dilatation with no respiratory change, and presence of respiratory change in flow velocities through the heart valves [47–49] (Figure 4).

Bedside echocardiography plays an important role in guiding pericardiocentesis. An extensive review from the Mayo Clinic on 1127 patients undergoing echocardiographically guided pericardiocentesis showed that the usual subxiphoid approach had been chosen in only 20% of cases after echocardiographic assessment. In most of the patients, pericardial puncture was performed using transthoracic puncture (apical/parasternal), and this reduced the complication rate of pericardiocentesis from 20% to only 4.7% [50].

## 5. Right Ventricle and Pulmonary Hypertension

**Figure 5:**
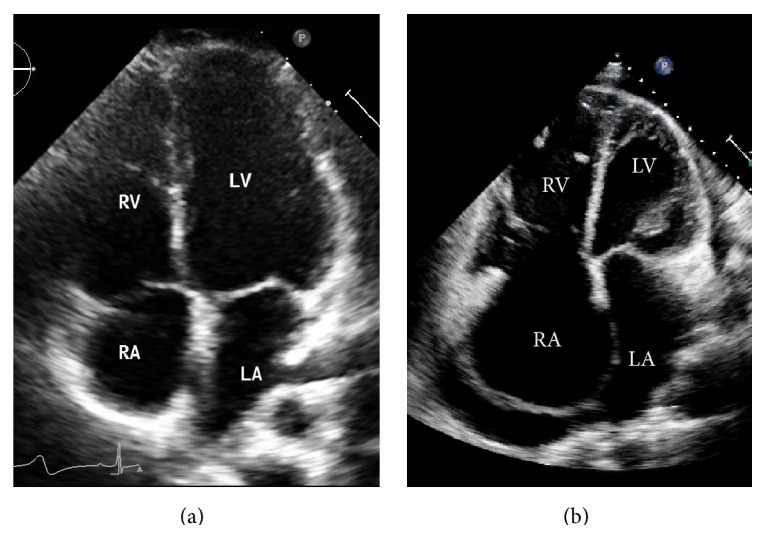
(a) Four-chamber apical view demonstrating normal heart. (b) Significant right chambers dilatation with straightened ventricular septum plus small pericardial effusion. LA: left atrium; LV: left ventricle; RA: right atrium; RV: right ventricle.

The right ventricular (RV) wall thickness and dimensions should be analyzed using all multiple views, the apical view being the most suitable for this analysis. The RV size is qualitatively analyzed and, in comparison to the LV size, is classified according to the RV and LV ratio as follows: normal, when the RV is smaller than the LV (approximately 60% of the LV size) and the RV apex is lower than the LV; slightly increased, when dilatation is present; however, RV is still smaller than LV; moderately increased, when the RV size is the same as LV; and markedly increased, when RV is larger than LV [46] (Figure 5).

The RV systolic function is assessed by nonechocardiographers only qualitatively, by visual estimation. Just like the assessment of LV function, ventricular wall motion and thickness should be analyzed. The classification of RV function is also similar to the LV's: normal, slightly reduced, moderately reduced or markedly reduced [46].

**Figure 6:**
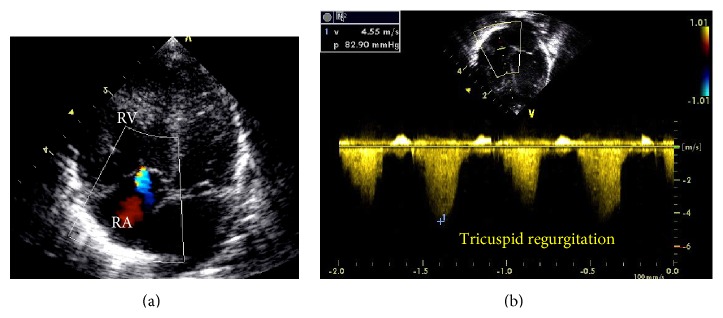
Newborn under invasive mechanical ventilation for hypoxemia in the first day of life. Apical view showing tricuspid regurgitation on color Doppler in blue (a) and on continuous wave Doppler (b). RV-RA gradient of 82 mmHg and the pulmonary artery systolic pressure is estimated at 92 mmHg (RV-RA gradient pressure added to the RA pressure).

Echocardiography also allows the estimative of pulmonary artery systolic pressure (PASP) in the presence of tricuspid regurgitation. Using the spectral curve of tricuspid regurgitation obtained by continuous wave Doppler, the pressure difference between RV and RA (gradient pressure) is calculated. The RV-RA gradient pressure added to the RA pressure is equivalent to PASP, when there is no RV outflow tract obstruction (Figure 6).

Pulmonary hypertension is present in several clinical situations in Pediatric Intensive Care, especially following cardiac surgery and in neonates. This makes its bedside diagnosis by the pediatrician both interesting and useful [51, 52].

## 6. Conclusion

Bedside echocardiography has become widespread in emergency and Intensive Care Units. It is a useful tool in the diagnosis and treatment of hemodynamic unstable adult and pediatric patients. Adequate training of pediatricians from Emergency and Intensive Care Units to perform focused echocardiography is feasible and provides improved care of severely ill children and thus should be encouraged.

## References

[1] Vieillard-Baron A., Prin S., Chergui K., Dubourg O., Jardin F. (2003). Hemodynamic instability in sepsis: bedside assessment by Doppler echocardiography. *American Journal of Respiratory and Critical Care Medicine*.

[2] Spencer K. T. (2015). Focused cardiac ultrasound: where do we stand?. *Current Cardiology Reports*.

[3] Colreavy F. B., Donovan K., Lee K. Y., Weekes J. (2002). Transesophageal echocardiography in critically ill patients. *Critical Care Medicine*.

[4] Croft L. B., Duvall W. L., Goldman M. E. (2006). A pilot study of the clinical impact of hand-carried cardiac ultrasound in the medical clinic. *Echocardiography*.

[5] Manasia A. R., Nagaraj H. M., Kodali R. B. (2005). Feasibility and potential clinical utility of goal-directed transthoracic echocardiography performed by noncardiologist intensivists using a small hand-carried device (SonoHeart) in critically ill patients. *Journal of Cardiothoracic and Vascular Anesthesia*.

[6] Ranjit S., Aram G., Kissoon N. (2014). Multimodal monitoring for hemodynamic categorization and management of pediatric septic shock: a pilot observational study. *Pediatric Critical Care Medicine*.

[7] Vincent J.-L., De Backer D. (2013). Circulatory shock. *The New England Journal of Medicine*.

[8] Klugman D., Berger J. T. (2011). Echocardiography as a hemodynamic monitor in critically ill children. *Pediatric Critical Care Medicine*.

[9] Mayo P. H., Beaulieu Y., Doelken P. (2009). American College of Chest Physicians/La Société de Réanimation de Langue Française statement on competence in critical care ultrasonography. *Chest*.

[10] Labovitz A. J., Noble V. E., Bierig M. (2010). Focused cardiac ultrasound in the emergent setting: a consensus statement of the American society of Echocardiography and American College of Emergency Physicians. *Journal of the American Society of Echocardiography*.

[11] Randazzo M. R., Snoey E. R., Levitt M. A., Binder K. (2003). Accuracy of emergency physician assessment of left ventricular ejection fraction and central venous pressure using echocardiography. *Academic Emergency Medicine*.

[12] DeCara J. M., Lang R. M., Koch R., Bala R., Penzotti J., Spencer K. T. (2003). The use of small personal ultrasound devices by internists without formal training in echocardiography. *European Journal of Echocardiography*.

[13] Pershad J., Myers S., Plouman C. (2004). Bedside limited echocardiography by the emergency physician is accurate during evaluation of the critically ill patient. *Pediatrics*.

[14] Spurney C. F., Sable C. A., Berger J. T., Martin G. R. (2005). Use of a hand-carried ultrasound device by critical care physicians for the diagnosis of pericardial effusions, decreased cardiac function, and left ventricular enlargement in pediatric patients. *Journal of the American Society of Echocardiography*.

[15] Vignon P., Dugard A., Abraham J. (2007). Focused training for goal-oriented hand-held echocardiography performed by noncardiologist residents in the intensive care unit. *Intensive Care Medicine*.

[16] Melamed R., Sprenkle M. D., Ulstad V. K., Herzog C. A., Leatherman J. W. (2009). Assessment of left ventricular function by intensivists using hand-held echocardiography. *Chest*.

[17] Vignon P., Mücke F., Bellec F. (2011). Basic critical care echocardiography: validation of a curriculum dedicated to noncardiologist residents. *Critical Care Medicine*.

[18] Longjohn M., Wan J., Joshi V., Pershad J. (2011). Point-of-care echocardiography by pediatric emergency physicians. *Pediatric Emergency Care*.

[19] Royse C. F., Haji D. L., Faris J. G., Veltman M. G., Kumar A., Royse A. G. (2012). Evaluation of the interpretative skills of participants of a limited transthoracic echocardiography training course (H.A.R.T.scan course). *Anaesthesia and Intensive Care*.

[20] Caronia J., Kutnick R., Sarzynski A., Panagopoulos G., Mahdavi R., Mina B. (2013). Focused transthoracic echocardiography performed and interpreted by medical residents in the critically Ill. *ICU Director*.

[21] Tanzola R. C., Walsh S., Hopman W. M., Sydor D., Arellano R., Allard R. V. (2013). Brief report: focused transthoracic echocardiography training in a cohort of Canadian anesthesiology residents: a pilot study. *Canadian Journal of Anesthesia*.

[22] Gaspar H. A., Morhy S. S., Lianza A. C. (2014). Focused cardiac ultrasound: a training course for pediatric intensivists and emergency physicians. *BMC Medical Education*.

[23] Amiel J.-B., Grümann A., Lhéritier G. (2012). Assessment of left ventricular ejection fraction using an ultrasonic stethoscope in critically ill patients. *Critical Care*.

[24] Razi R., Estrada J. R., Doll J., Spencer K. T. (2011). Bedside hand-carried ultrasound by internal medicine residents versus traditional clinical assessment for the identification of systolic dysfunction in patients admitted with decompensated heart failure. *Journal of the American Society of Echocardiography*.

[25] Ünlüer E. E., Karagöz A., Akoğlu H., Bayata S. (2014). Visual estimation of bedside echocardiographic ejection fraction by emergency physicians. *Western Journal of Emergency Medicine*.

[26] Margossian R., Schwartz M. L., Prakash A. (2009). Comparison of echocardiographic and cardiac magnetic resonance imaging measurements of functional single ventricular volumes, mass, and ejection fraction (from the Pediatric Heart Network Fontan Cross-Sectional Study). *American Journal of Cardiology*.

[27] Gazit A. Z., Cooper D. S. (2011). Emerging technologies. *Pediatric Critical Care Medicine*.

[28] Cecconi M., de Backer D., Antonelli M. (2014). Consensus on circulatory shock and hemodynamic monitoring. Task force of the European Society of Intensive Care Medicine. *Intensive Care Medicine*.

[29] Dellinger R. P., Levy M. M., Rhodes A. (2013). Surviving sepsis campaign: international guidelines for management of severe sepsis and septic shock, 2012. *Intensive Care Medicine*.

[30] Dinh V. A., Ko H. S., Rao R. (2012). Measuring cardiac index with a focused cardiac ultrasound examination in the ED. *American Journal of Emergency Medicine*.

[31] Boyd J. H., Forbes J., Nakada T.-A., Walley K. R., Russell J. A. (2011). Fluid resuscitation in septic shock: a positive fluid balance and elevated central venous pressure are associated with increased mortality. *Critical Care Medicine*.

[32] Raux O., Spencer A., Fesseau R. (2012). Intraoperative use of transoesophageal doppler to predict response to volume expansion in infants and neonates. *British Journal of Anaesthesia*.

[33] Lukito V., Djer M. M., Pudjiadi A. H., Munasir Z. (2012). The role of passive leg raising to predict fluid responsiveness in pediatric intensive care unit patients. *Pediatric Critical Care Medicine*.

[34] Durand P., Chevret L., Essouri S., Haas V., Devictor D. (2008). Respiratory variations in aortic blood flow predict fluid responsiveness in ventilated children. *Intensive Care Medicine*.

[35] Michard F., Teboul J.-L. (2002). Predicting fluid responsiveness in ICU patients: a critical analysis of the evidence. *Chest*.

[36] Gan H., Cannesson M., Chandler J. R., Ansermino J. M. (2013). Predicting fluid responsiveness in children: a systematic review. *Anesthesia & Analgesia*.

[37] Haines E. J., Chiricolo G. C., Aralica K. (2012). Derivation of a pediatric growth curve for inferior vena caval diameter in healthy pediatric patients: brief report of initial curve development. *Critical Ultrasound Journal*.

[38] Feissel M., Michard F., Faller J.-P., Teboul J.-L. (2004). The respiratory variation in inferior vena cava diameter as a guide to fluid therapy. *Intensive Care Medicine*.

[39] Barbier C., Loubières Y., Schmit C. (2004). Respiratory changes in inferior vena cava diameter are helpful in predicting fluid responsiveness in ventilated septic patients. *Intensive Care Medicine*.

[40] Mandeville J. C., Colebourn C. L. (2012). Can transthoracic echocardiography be used to predict fluid responsiveness in the critically ill patient? A systematic review. *Critical Care Research and Practice*.

[41] Choi D. Y., Kwak H. J., Park H. Y., Kim Y. B., Choi C. H., Lee J. Y. (2010). Respiratory variation in aortic blood flow velocity as a predictor of fluid responsiveness in children after repair of ventricular septal defect. *Pediatric Cardiology*.

[42] Byon H.-J., Lim C.-W., Lee J.-H. (2013). Prediction of fluid responsiveness in mechanically ventilated children undergoing neurosurgery. *British Journal of Anaesthesia*.

[43] Feissel M., Michard F., Mangin I., Ruyer O., Faller J.-P., Teboul J.-L. (2001). Respiratory changes in aortic blood velocity as an indicator of fluid responsiveness in ventilated patients with septic shock. *Chest*.

[44] de Souza Neto E. P., Grousson S., Duflo F. (2011). Predicting fluid responsiveness in mechanically ventilated children under general anaesthesia using dynamic parameters and transthoracic echocardiography. *British Journal of Anaesthesia*.

[45] Renner J., Broch O., Duetschke P. (2012). Prediction of fluid responsiveness in infants and neonates undergoing congenital heart surgery. *British Journal of Anaesthesia*.

[46] Otto C. M. (2009). *Fundamentos de Ecocardiografia Clínica*.

[47] Goodman A., Perera P., Mailhot T., Mandavia D. (2012). The role of bedside ultrasound in the diagnosis of pericardial effusion and cardiac tamponade. *Journal of Emergencies, Trauma and Shock*.

[48] Tsang T. S. M., Oh J. K., Seward J. B. (1999). Diagnosis and management of cardiac tamponade in the era of echocardiography. *Clinical Cardiology*.

[49] Tsang T. S. M., Oh J. K., Seward J. B., Tajik A. J. (2000). Diagnostic value of echocardiography in cardiac tamponade. *Herz*.

[50] Tsang T. S. M., Enriquez-Sarano M., Freeman W. K. (2002). Consecutive 1127 therapeutic echocardiographically guided pericardiocenteses: clinical profile, practice patterns, and outcomes spanning 21 years. *Mayo Clinic Proceedings*.

[51] Jain A., McNamara P. J. (2015). Persistent pulmonary hypertension of the newborn: advances in diagnosis and treatment. *Seminars in Fetal and Neonatal Medicine*.

[52] Ofori-Amanfo G., Cheifetz I. M. (2013). Pediatric postoperative cardiac care. *Critical Care Clinics*.

